# Supplementary material for: Research on the impact of COVID-19 on Chinese small and medium-sized enterprises: Evidence from Beijing
Source: PLoS One. 2021 Dec 9;16(12):e0257036. doi: 10.1371/journal.pone.0257036 (PMC8659340; doi:10.1371/journal.pone.0257036)
Supplement: S1 File — (DOCX) [file pone.0257036.s001.docx]

**Questionnaire of impact of COVID-19 on small and medium-sized enterprises**

Dear sir:

In order to further explore the factors affecting the development of small and medium-sized enterprises during COVID-19, the School of Economics and Management of China University of Petroleum now conducts a questionnaire survey on the relevant situation. We are very eager to truly understand the impact of the epidemic on small and medium-sized enterprises through your sincere help.

This questionnaire includes three parts, please complete the questionnaire in a busy schedule. The data from this study are used for scientific statistical analysis without any commercial use. At the same time, we promise to keep your personal information strictly confidential. Thank you.

**Part 1**

Please fill according to your actual situation.

Sex: male female

Age: 20-30 30-40 40-50 50-60

Industry: Processing and manufacturing industry

Logistics and transportation & Wholesale trade

Retail and Services Industry (Education, incubation platform)

Agriculture, forestry, animal husbandry and fishery

High-technology (including software and Internet)

Catering, accommodation, entertainment and cultural tourism

E-commerce Construction industry Others

Career: Employee Department Director

Manager　 General Manager and above

Number of employees: Less than 20 21-50 51-100

101-200 201-300 301-500

**Part 2**

Please select a number that will best represent your idea. **The smaller number you choose, the more you agree with the idea, and the larger number you choose, the more you disagree with the idea (1 representative completely agree and 7 representative completely disagree).**

| Factor one: Finance | | | | | | | | |
| --- | --- | --- | --- | --- | --- | --- | --- | --- |
| No. | Content | 1 | 2 | 3 | 4 | 5 | 6 | 7 |
| 1 | Due to COVID-19, business operating revenue decreased significantly. |  |  |  |  |  |  |  |
| 2 | Due to COVID-19, corporate sales profits decreased significantly. |  |  |  |  |  |  |  |
| 3 | During COVID-19, the ability of enterprises to repay external debt deteriorated. |  |  |  |  |  |  |  |
| 4 | During COVID-19, enterprise cash and other emergency liquidity stock were significantly tight. |  |  |  |  |  |  |  |
| 5 | During COVID-19, financing requirements increased significantly to relieve operating pressure. |  |  |  |  |  |  |  |
| Factor two: Market | | | | | | | | |
| No. | Content | 1 | 2 | 3 | 4 | 5 | 6 | 7 |
| 6 | During COVID-19, supply of materials needed by the enterprise was significantly reduced. |  |  |  |  |  |  |  |
| 7 | During COVID-19, consumers' demand for goods and services provided by the enterprise was significantly reduced. |  |  |  |  |  |  |  |
| 8 | During COVID-19, the price at which the company sold goods and provided services was significantly reduced. |  |  |  |  |  |  |  |
| 9 | Compared with the time before COVID-19, there was a backlog of inventory in the enterprise. |  |  |  |  |  |  |  |
| 10 | Compared with the time before COVID-19, the export business of the enterprise decreased significantly, and the export defaults increased significantly. |  |  |  |  |  |  |  |
| Factor three: Employee | | | | | | | | |
| No. | Content | 1 | 2 | 3 | 4 | 5 | 6 | 7 |
| 11 | Due to COVID-19, it was very difficult for the enterprise to recruit workers. |  |  |  |  |  |  |  |
| 12 | Due to COVID-19, corporate layoffs increased significantly. |  |  |  |  |  |  |  |
| 13 | Due to COVID-19, the turnover rate of enterprise employees increased significantly, and the employee loyalty was significantly reduced. |  |  |  |  |  |  |  |
| 14 | During COVID-19, the working time of employees and the work efficiency were both significantly reduced. |  |  |  |  |  |  |  |
| 15 | During COVID-19, the online office of staff brought great inconvenience to enterprise management. |  |  |  |  |  |  |  |
| Factor four: Cost | | | | | | | | |
| No. | Content | 1 | 2 | 3 | 4 | 5 | 6 | 7 |
| 16 | During COVID-19, the price of raw materials purchased by the enterprise increased significantly. |  |  |  |  |  |  |  |
| 17 | During COVID-19, staff epidemic prevention materials and epidemic prevention allowance increased labor costs of the enterprise. |  |  |  |  |  |  |  |
| 18 | During COVID-19, costs of enterprise online office and transportation of goods increased significantly. |  |  |  |  |  |  |  |
| 19 | Due to COVID-19, the enterprise strengthened the epidemic prevention work, so that costs of management and training significantly increased. |  |  |  |  |  |  |  |
| 20 | Due to the impact of COVID-19 on raw material supply and employee stability, the average production cycle and service supply cycle of a single product were significantly extended. |  |  |  |  |  |  |  |
| Factor five: Policy | | | | | | | | |
| No. | Content | 1 | 2 | 3 | 4 | 5 | 6 | 7 |
| 21 | During COVID-19, the government tax relief policy had a great positive impact on the survival and development of small and medium-sized enterprises. |  |  |  |  |  |  |  |
| 22 | During COVID-19, the government employment subsidy policy had a great positive impact on the survival and development of small and medium-sized enterprises. |  |  |  |  |  |  |  |
| 23 | During COVID-19, the government operational subsidy policy had a great positive impact on the survival and development of small and medium-sized enterprises. |  |  |  |  |  |  |  |
| 24 | During COVID-19, the government policy on rent relief for commercial property had a great positive impact on the survival and development of small and medium-sized enterprises. |  |  |  |  |  |  |  |
| 25 | During COVID-19, bank interest-free loans and loan delay repayment policy had a great positive impact on the survival and development of small and medium-sized enterprises. |  |  |  |  |  |  |  |

**Note: The smaller number you choose, the more you agree with the idea, and the larger number you choose, the more you disagree with the idea (1 representative completely agree and 7 representative completely disagree).**

| Impact of COVID-19 on SMEs | | | | | | | | |
| --- | --- | --- | --- | --- | --- | --- | --- | --- |
| No. | Content | 1 | 2 | 3 | 4 | 5 | 6 | 7 |
| 26 | COVID-19 had a significant impact on our business. |  |  |  |  |  |  |  |
| 27 | The impact of COVID-19 on our business will continue for a while. |  |  |  |  |  |  |  |
| 28 | COVID-19 had a great impact on the small and medium-sized enterprises around me. |  |  |  |  |  |  |  |

**Part 3**

Please answer the questions based on your understanding of the survival and development conditions of SMEs during COVID-19.

1、In addition to the factors mentioned in this questionnaire, what are the other factors of the impact of COVID-19 on small and medium-sized enterprises?
