# Supplementary material for: Research on the impact of COVID-19 on Chinese small and medium-sized enterprises: Evidence from Beijing
Source: PLoS One. 2021 Dec 9;16(12):e0257036. doi: 10.1371/journal.pone.0257036 (PMC8659340; doi:10.1371/journal.pone.0257036)
Supplement: S2 File — (DOCX) [file pone.0257036.s002.docx]

**疫情对中小企业影响因素分析问卷设计**

尊敬的阁下：您好！

为进一步探究疫情期间影响中小企业发展的因素，中国石油大学工商管理学院现就有关情况进行问卷调查。我们非常渴望通过您的真诚帮助，为我们真实了解疫情对中小企业的影响因素提供机会。

本问卷共包括三个部分，烦请您在百忙之中完成这份问卷。本研究的数据只用于科学统计分析，没有任何商业用途。同时我们承诺，将对您个人资料严格保密，谢谢。

**第一部分**

请根据您的实际情况打钩。

性别： 男 女

年龄： 20-30 30-40 40-50 50-60

行业：加工制造业 物流运输批发贸易

零售与服务业(教育、孵化平台) 　 农林牧副渔业

高科技(含软件、互联网) 餐饮住宿娱乐文化旅游

电商 建筑业 其他

职位：普通员工 部门主管 经理或总监　 总经理及以上

企业员工数量：20人以下 21~50人 51~100人

101~200人 201~300人 301~500人

**第二部分**

请选择最能够代表您想法的一个数字。**您选的数字越小代表您越同意前述观点，您选的数字越大代表您越不同意前述观点（1表示完全同意，7表示完全不同意）。**

| 因素1：财务现金流 | | | | | | | | |
| --- | --- | --- | --- | --- | --- | --- | --- | --- |
| 序号 | 内容 | 1 | 2 | 3 | 4 | 5 | 6 | 7 |
| 1 | 由于新冠疫情影响，企业营业收入明显减少。 |  |  |  |  |  |  |  |
| 2 | 由于新冠疫情影响，企业销售利润明显减少。 |  |  |  |  |  |  |  |
| 3 | 疫情期间企业偿还外债能力变差。 |  |  |  |  |  |  |  |
| 4 | 疫情期间企业现金等应急流动资金明显紧张。 |  |  |  |  |  |  |  |
| 5 | 疫情期间企业为缓解经营压力，融资需求明显增加。 |  |  |  |  |  |  |  |
| 因素2：市场供需 | | | | | | | | |
| 序号 | 内容 | 1 | 2 | 3 | 4 | 5 | 6 | 7 |
| 6 | 疫情期间，企业经营业务所需物资材料的供给量明显减少。 |  |  |  |  |  |  |  |
| 7 | 疫情期间，消费者对企业提供的商品与劳务的需求明显减少。 |  |  |  |  |  |  |  |
| 8 | 疫情期间，企业销售商品及提供劳务的价格明显降低。 |  |  |  |  |  |  |  |
| 9 | 与疫情前相比，企业存货明显积压。 |  |  |  |  |  |  |  |
| 10 | 与疫情前相比，企业出口业务明显减少，出口违约大幅增加。 |  |  |  |  |  |  |  |
| 因素3：人员流动 | | | | | | | | |
| 序号 | 内容 | 1 | 2 | 3 | 4 | 5 | 6 | 7 |
| 11 | 由于新冠疫情的影响，企业招工十分困难。 |  |  |  |  |  |  |  |
| 12 | 由于新冠疫情的影响，企业裁员明显增加。 |  |  |  |  |  |  |  |
| 13 | 由于新冠疫情的影响，企业员工离职率明显增加，员工忠诚度明显降低。 |  |  |  |  |  |  |  |
| 14 | 疫情期间，员工工作时长大幅减少，工作效率明显降低。 |  |  |  |  |  |  |  |
| 15 | 疫情期间，员工线上办公为企业管理带来较大的不便。 |  |  |  |  |  |  |  |
| 因素4：成本 | | | | | | | | |
| 序号 | 内容 | 1 | 2 | 3 | 4 | 5 | 6 | 7 |
| 16 | 疫情期间，企业采购的原材料价格明显提高。 |  |  |  |  |  |  |  |
| 17 | 疫情期间，员工防疫物资、防疫津贴等增加了企业的用工成本。 |  |  |  |  |  |  |  |
| 18 | 疫情期间，企业线上办公及运输商品的成本明显增加。 |  |  |  |  |  |  |  |
| 19 | 由于新冠疫情，企业加强防疫工作，使管理及培训成本明显增加。 |  |  |  |  |  |  |  |
| 20 | 由于疫情对原材料供给及员工稳定性的冲击，企业单一产品平均生产周期与服务提供周期明显延长。 |  |  |  |  |  |  |  |
| 因素5：政府政策 | | | | | | | | |
| 序号 | 内容 | 1 | 2 | 3 | 4 | 5 | 6 | 7 |
| 21 | 疫情期间，政府税收减免政策对中小企业的生存和发展有较大的积极影响。 |  |  |  |  |  |  |  |
| 22 | 疫情期间，政府就业补贴政策对中小企业的生存和发展有较大的积极影响。 |  |  |  |  |  |  |  |
| 23 | 疫情期间，政府运营补贴政策对中小企业的生存和发展有较大的积极影响。 |  |  |  |  |  |  |  |
| 24 | 疫情期间，政府对商业物业租金减免的政策对中小企业的生存和发展有较大的积极影响。 |  |  |  |  |  |  |  |
| 25 | 疫情期间，银行免息贷款及贷款延缓偿还政策对中小企业的生存和发展有较大的积极影响。 |  |  |  |  |  |  |  |

**注：您选的数字越小代表您越同意前述观点，您选的数字越大代表您越不同意前述观点（1表示完全同意，7表示完全不同意）。**

| 疫情影响 | | | | | | | | |
| --- | --- | --- | --- | --- | --- | --- | --- | --- |
| 序号 | 内容 | 1 | 2 | 3 | 4 | 5 | 6 | 7 |
| 26 | 新冠疫情对我们企业有明显的影响。 |  |  |  |  |  |  |  |
| 27 | 新冠疫情对我们企业影响还将持续一段时间。 |  |  |  |  |  |  |  |
| 28 | 据我了解新冠疫情对我周围的中小企业产生了较大影响。 |  |  |  |  |  |  |  |

**第三部分**

请根据您对疫情期间中小企业生存发展状况的了解回答问题。

1、您认为除了本问卷提到的因素外，还有哪些因素是疫情对中小企业的影响因素?
