# Supplementary material for: Research on the impact of COVID-19 on Chinese small and medium-sized enterprises: Evidence from Beijing
Source: PLoS One. 2021 Dec 9;16(12):e0257036. doi: 10.1371/journal.pone.0257036 (PMC8659340; doi:10.1371/journal.pone.0257036)
Supplement: Attachment — Submitted filename: Detailed Response to Reviewer two.docx [file pone.0257036.s003.docx]

Thank you so much for the reviewers’ comments concerning our manuscript entitled “Research on the Environmental Impact of Tidal Power Generation in China”. Those comments are all valuable and very helpful for revising and improving our manuscript, as well as the important guiding significance to our study. We have studied comments carefully and have made correction which we hope meet with approval. Revised portion are marked in red in the paper. The main corrections in the paper and the responds to the reviewer’s comments are as flowing:

Responds to the reviewer’s comments:

**Reviewer #2:**

1. 1.1, the Importance of SME-s is instead belonging to Literature review, so I recommend shifting it there. 1.2 will be enough for introduction, extended by describing the research goals and the context better.

***Response:*** Thank you so much for your comment. We have moved 1.1 to Literature review and revised the subtitle of 1.2. Revised portions are marked in red in the paper.

2. Literature review exists; however, it is unusually detailed articulated, and in some parts, I feel it is instead a chapter of a theoretical book or university booklet. More emphasis should be set on the critical and analytical approach while processing the literature sources.

***Response:*** Thank you so much for your comment. Following your suggestion, we have revised the literature review. We add the new chapter "Analytical approach" in literature review. Please review it. Revised portions are marked in red in the paper.

3. While there is a methodology chapter, we can see an unusual chapter named the "Hypothesis and construct" chapter, indicating 15 (!) hypotheses. First of all, in my opinion, it should be merged with the methodology chapter.

***Response:*** Thank you so much for your comment. Following your suggestion, we have merged "Hypothesis and analysis" chapter with "methodology" chapter. Please review it. Revised portions are marked in red in the paper.

4. On the other hand, 15 hypotheses are just too much. As not all of them belong to the category of accurate and well-defined hypotheses, please merge or cancel a few.

***Response:*** Thank you so much for your comment. As there is no well-established SEM that has existed in areas studying the impact of COVID-19 on enterprises, we need to construct the exploratory structural equation model instead of confirmatory structural equation model. Initially we need to consider all possible assumptions that might exist and revise the model after comparison with actual observed data to get the best-fit model^[1]^. Consequently, the initial hypotheses seem to be too much.

5. In addition, it is just not clear on what basis these hypotheses are defined.

***Response:*** Thank you so much for your comment. We reconstruct chapter 3.1.2 "Hypothesis" and add the theoretical basis for these hypothetical definitions. Please review it.

6. Regarding the results, why these are valid, however, there is a disproportion regarding the structure as it is too short. While the methodology is explained on several pages, the results are on net 2 pages, without a real explanation.

***Response:*** Thank you so much for your comment. We have rewritten the chapter “Results”. Revised portion are marked in red in the paper. Please review it.

7. There are no limitations indicated in the text; it should be done in the introduction, methodology, or conclusions.

***Response:*** Thank you so much for your comment. We add the new chapter "Limitations and future research" before chapter "Conflicts of Interest". Please review it.

Thank you so much for your comments. I really learn a lot for your comments. All comments are very valuable and helpful for revising and improving our manuscript, as well as the important guiding significance to our study.

References:

[1] Jöreskog, K.G. & Sörbom, D. (1996). LISREL 8 User's reference guide. Chicago: Scientific Software.
