# Supplementary material for: Research on the impact of COVID-19 on Chinese small and medium-sized enterprises: Evidence from Beijing
Source: PLoS One. 2021 Dec 9;16(12):e0257036. doi: 10.1371/journal.pone.0257036 (PMC8659340; doi:10.1371/journal.pone.0257036)
Supplement: Attachment — Submitted filename: Detailed Response to Reviewer two.docx [file pone.0257036.s004.docx]

Thank you so much for the your comment concerning our manuscript entitled “Research on the impact of COVID-19 on Chinese small and medium-sized enterprises : Evidence from Beijing”. The comment is valuable and very helpful for revising and improving our manuscript, as well as the important guiding significance to our study. We have studied the comment carefully and have made correction which we hope meet with approval. Revised portion are marked in red in the paper. The main corrections in the paper and the respond to your comment is as flowing:

Responds to the reviewer’s comment:

**Reviewer #2:**

1. The paper is much better in its current version, however, I still have concerns regarding the large number of hypotheses, I still recommend to lower the number of hypotheses, deleting and merging some.

***Response:*** Thank you so much for your comment. We have deleted and merged some hypotheses and restructured the model. Revised portions are marked in red in the paper.

Thank you again for your comment. I really learn a lot for your comment. The comment is very valuable and helpful for revising and improving our manuscript, as well as the important guiding significance to our study.
